# OPTIMIZING MEDICATION RECONCILIATION AMONG OLDER ADULTS DURING COVID-19 INFECTION TREATMENT WITH PAXLOVID

**DOI:** 10.1093/geroni/igac059.2976

**Published:** 2022-12-20

**Authors:** John Chen, Tarah Browne

**Affiliations:** Kaiser Permanente Med Group/Southern California, Harbor City, California, United States; Kaiser Permanente Med Group/Southern California, Harbor City, California, United States

## Abstract

COVID-19 infection treatment (CIT) has reframed optimizing medication reconciliation in older adults. In December 2021, FDA authorized Emergency Use Authorization of Paxlovid (Nirmatrelvir/Ritonavir), protease inhibitor blocking SARS-CoV-2 replication, to reduce severe COVID-19 and mortality in high-risk patients1. Pharmacodynamic and pharmacokinetic changes in geriatric population warrant a stepwise, patient-centered approach for prescribing Paxlovid by reviewing - 1) drug-drug interactions with current medications2, 2) renal dosing, 3) risks-benefits-alternatives, 4) adverse drug events (ADE), i.e. polypharmacy and prescribing cascade. Case A – Community-dwelling 65-year-old Hispanic male history of paroxysmal ventricular tachycardia and end-stage renal disease (ESRD) requiring CIT- 1) Paxlovid contraindication with Amiodarone2 2) ESRD – not candidate 3) risks outweigh benefits, 4) safer alternatives with less ADE. Case B – Long term care 80-year-old Hispanic female history of stroke, type 2 diabetes mellitus and dementia requiring CIT -1) Paxlovid contraindication with Atorvastatin2 (Atorvastatin held during Paxlovid treatment then restarted at lower dose “deprescribing”), 2) estimated glomerular filtration rate 46 with renal dosing – Paxlovid 150mg/100mg, 3) benefits outweigh risks, 4) monitored ADE in controlled setting. Optimizing medication reconciliation during CIT in older patients can decrease preventable ADE. Further research is essential to align CIT efficacy with the fundamental geriatric principles, including shared decision-making, long-term prognosis, and life care planning. 1Najjar-Debbiny R, Gronich N, Weber G, Khoury J, Amar M, Stein N, Goldstein LH, Saliba W. Effectiveness of Paxlovid in Reducing Severe COVID-19 and Mortality in High Risk Patients. Clin Infect Dis. 2022 Jun 2:ciac443. 2Lexicomp Drug Interaction Checker. Accessed August 6, 2022.

